# Dam Safety Evaluation Method after Extreme Load Condition Based on Health Monitoring and Deep Learning

**DOI:** 10.3390/s23094480

**Published:** 2023-05-04

**Authors:** Jintao Song, Yunhe Liu, Jie Yang

**Affiliations:** School of Water Resources and Hydro-Electric Engineering, Xi’an University of Technology, Xi’an 710048, China

**Keywords:** dam, structural health monitoring, monitoring model, extreme load, safety evaluation, deep learning

## Abstract

The safety operation of dams after extreme load is an important frontier research topic in the field of dam engineering. The dam health monitoring provides a reliable data basis for a safety evaluation after extreme loads. This study proposes a novel data-driven fusion model for a dam safety evaluation after extreme load based on monitoring data derived by sensors. First, the relationship between dam environmental quantity and effect quantity is deeply excavated based on bidirectional long short-term memory (BiLSTM) network, which is a deeply improved LSTM model. Aiming at the parameter optimization problem of BiLSTM model, sparrow search algorithm (SSA), which is an advanced optimization algorithm, is integrated. Second, conducting the constructed SSA-BiLSTM model to estimate the change law of dam effect quantity after the extreme load. Finally, the Mann–Whitney U-test theory is introduced to establish the evaluation criterion of the dam safety state. Project case shows that the multiple quantitative prediction accuracy evaluation indicators of the proposed method are significantly superior to the comparison method, with mean absolute percentage error (MAPE) and mean absolute error (MAE) values decreasing by 30.5% and 27.8%, respectively, on average. The proposed model can accurately diagnose the dam safety state after the extreme load compared with on-site inspection results of the engineering department, which provides a new method for dam safety evaluation.

## 1. Introduction

The dam project has played an important role in economic development worldwide, and the safety operation of the project is of great significance [1,2]. In addition to the conventional water and temperature loads, these projects are also subjected to extreme loads [3]. In recent years, serious natural disasters, such as strong earthquakes and rainstorms, happened more frequently around the world. For example, the United States was affected by the Ida typhoon in September 2021, resulting in an extreme rainfall, which had the largest rainfall record in history. In August 2021, Henan and other provinces in China suffered extreme rainfall disasters, with a daily maximum rainfall of 1000 mm. In January 2022, a strong earthquake occurred in Qinghai, with a magnitude of 6.9. The occurrence of extreme loads has a serious impact on the safety of dams [4,5].

Some scholars have carried out systematic research on the evaluation of dam safety status under extreme loads. For strong earthquake loads, Zou [6,7] systematically analyzed the safety problems and analysis methods under strong earthquakes in the process of dam construction and operation. Lin and Gao [8,9] discussed the failure mechanism and seismic performance of dams from the aspects of dam destructive behavior, acceleration response, stress, and deformation during the Wenchuan earthquake. Karalar [10,11] evaluated the influence of strong earthquakes on the nonlinear seismic settlement and shear strain behavior of Oroville dam in the United States based on the finite difference method. Nasiri [12] analyzed the influence law of different seismic recording methods on dam dynamic response and verified the feasibility of wavelet decomposition recording method. For the rainstorm loads, Choi [13] studied the aging problem of dams under the condition of heavy rainfall and put forward the engineering measures of danger removal and reinforcement according to different aging degrees. Henn [14] analyzed the impact of heavy rainfall caused by a super typhoon in the United States in 2017 on the Oroville dam spillway and systematically summarized the loss of life and property caused by the accident. Chen [15] proposed a method to determine the critical precipitation of the dam under the action of heavy rainfall, which can evaluate the risk of dam failure on the basis of probability and provide early warning in case of emergency. It can be concluded that some scholars have conducted systematic and deep research on the safety of dams under different extreme loads and obtained a large number of research results to guide engineering operation. However, due to the variety of extreme loads, including both dynamic and static loads, the dam operation status under extreme loads is complex, so it is difficult to construct a unified safety evaluation method under different extreme loads. 

Structural health monitoring is an important research direction in the field of dam safety evaluation [16,17]. By constructing the nonlinear relationship between environmental load data and dam effect quantity data derived from the dam sensors, the safety behavior of the dam can be evaluated quantitatively. Due to the short occurrence time of the extreme load, there are limitations in evaluating the dam safety state under the action of the extreme load caused by the few amounts of monitoring data. However, the monitoring data after the end of the extreme load is sufficient. It can be concluded from many operation reports that the number of dams that crashed during the action of extreme loads is relatively small, but the dam may partly have abnormal conditions, such as cracking and aging after the action of extreme loads [18,19]. If it cannot be detected in time, the risk degree may expand sharply, resulting in partial or overall damage to the structure [20,21]. Therefore, aiming at the problem of dam safety state evaluation after the end of extreme load condition, it is an important issue to establish a unified safety state evaluation model based on dam monitoring data.

The development of dam safety monitoring model has experienced two stages: statistical modeling and artificial intelligence modeling. In recent years, deep learning, which is the frontier field of artificial intelligence has been widely used in dam monitoring model, especially the long and short-term memory network (LSTM) [22]. However, LSTM is a unidirectional neural network, which cannot fully extract the reverse transmission characteristics of information. Bi-directional long and short-term memory (BiLSTM), which is a combined of forward and backward LSTM, can better obtain the connection relationship between input and output information [23,24]. The parameter selection is a common problem in the prediction performance of deep learning algorithm, which has a significant impact on the prediction performance of the model. Sparrow search optimization algorithm (SSA) is an advanced bionic intelligent optimization algorithm proposed in 2020 and has been well applied in the field of model parameter optimization [25]. Therefore, the BiLSTM deep learning model and SSA optimization algorithm can be cross fused to predict the status of the dam before the extreme load with high precision.

Consequently, aiming at the dam safety evaluation after the end of extreme load, the BiLSTM advanced artificial intelligence method is introduced. This method is improved on the typical LSTM methods and constructed a two-way LSTM network which can deeply mine the changing rule of the dam monitoring data. In order to further improve the evaluation accuracy of BiLSTM, the SSA optimization algorithm is combined and the dam safety evaluation criteria after extreme load is constructed by U-test theory. The U-test theory is a classic discrimination method for evaluating whether two sets of data conform to the same law. The fusion evaluation method focuses on analyzing the consistency of changes in dam monitoring data before and after the occurrence of extreme loads, which is different from traditional extreme load evaluation methods. Finally, taking a dam project in China as the case study, the dam has experienced extreme loads, such as Wenchuan earthquake in 2008 and heavy rainfall in 2021, which can illustrate the effectiveness and rationality of the proposed method.

## 2. Methods

### 2.1. Bi-Directional Long Short-Term Memory (BiLSTM)

**Figure 1:**
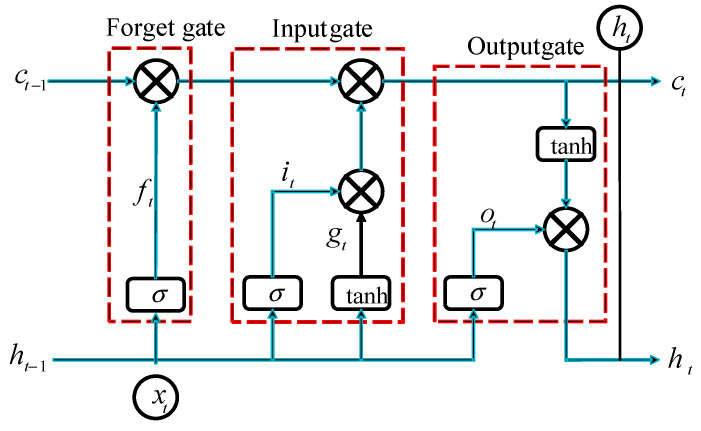
LSTM internal unit structure diagram.

The long short-term memory neural network (LSTM) is a widely used deep learning model in recent years. LSTM neural network contains a complex dynamic structure, including three gate structures (input gate, forgetting gate, and output gate) [26]. The internal unit structure of LSTM neural network is shown in Figure 1.

The calculation formula of LSTM neural network is as follows:

(1) $$\{\begin{array}{l} i_{t}=\sigma (W_{xi}x_{i}+W_{hi}h_{i-1}+W_{ci}c_{i-1}+b_{i})\\ f_{t}=\sigma (W_{xf}x_{t}+W_{hf}h_{t-1}+W_{cf}c_{t-1}+b_{f})\\ c_{t}=f_{t}h_{t-1}+i_{t}\tanh(W_{xc}x_{t}+W_{hc}h_{t-1}+b_{c})\\ o_{t}=\sigma (W_{xo}x_{t}+W_{ho}h_{t-1}+W_{co}c_{t}+b_{o})\\ h_{t}=o_{t}\tanh(c_{t}) \end{array}$$

where $i_{t}$, $f_{t}$, $c_{t}$, and $o_{t}$ are input gate, forgetting gate, updated cell state, and output gate, respectively; $x_{t}$ is input information; $h_{t}$ is the output information; W is the weight coefficient; $b_{i}$, $b_{f}$, $b_{c}$, and $b_{o}$ are the offset, respectively; σ and tanh are sigmoid functions and hyperbolic tangent functions, respectively.

LSTM model has good prediction ability for nonlinear time series, but the model is a one-way transmission process that the predicted value at later time has no influence on the predicted value of current time. For dam intelligent prediction model, it should comprehensively consider the two-way dynamic relationship between input and output at different times and use the new monitoring value to reverse correct the prediction value to improve the prediction effect. Bidirectional long- and short-term neural network (BiLSTM) is an advanced two-way deep learning neural network improved by the LSTM model and can achieve better prediction results than LSTM model [27,28].

**Figure 2:**
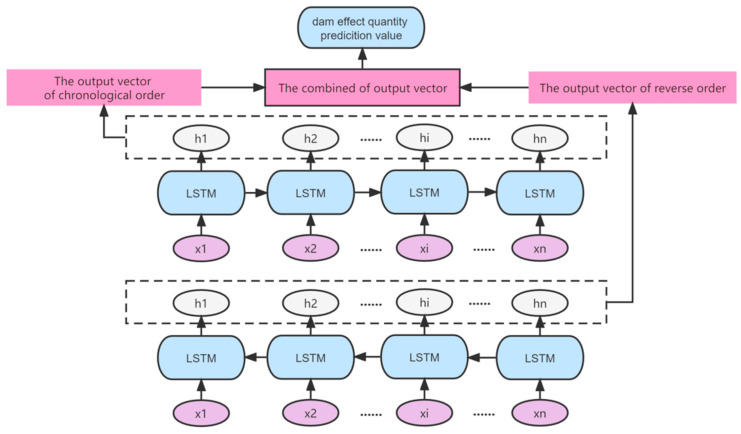
BiLSTM internal unit structure diagram.

BiLSTM neural network is constructed by two LSTM layers in opposite directions, one layer input the data in chronological order from the beginning to the end, and the other layer input the data in reverse order from the end to the beginning. This pair of hidden layers with opposite directions are finally connected to the output value. Therefore, the prediction performance of BiLSTM is better than that of ordinary LSTM or recurrent neural network (RNN). The network structure of BiLSTM is shown in Figure 2. For the input, $x_{i}(i=1,2\cdots n)$, the BiLSTM model adopts the forward LSTM to perform forward recursion, which takes $x_{1}$ as the input data at the start time. Meanwhile, the reverse LSTM takes $x_{n}$ as the input data at the start time. In the process of network training, the dam monitoring data sequence is input into the forward LSTM and reverse LSTM at the same time to obtain the output data $h_{t}$ at time t in two directions. The two-output data are combined to obtain the final output y. The BiLSTM model is expressed as:

(2) $$\left\{\begin{array}{l} h_{t}=\alpha h_{t}^{f}+\beta h_{t}^{b}\\ y_{t}=\sigma (h_{t}) \end{array}\right.$$

where $h_{t}^{f}$ and $h_{t}^{b}$ are the output of the hidden layer in the forward and reverse LSTM model at time, respectively; t, α, and β are the weight coefficient, respectively; and α+β=1. σ is the sigmoid activate function and $\sigma (x)=\frac{1}{1+\exp(-x)}$.

### 2.2. Sparrow Search Algorithm (SSA)

Sparrow search algorithm (SSA) is a new bionic optimization algorithm proposed in 2020 [29,30]. Its inspiration mainly comes from sparrows’ group intelligence, foraging, and anti-predator behavior. It has the advantages of fast convergence speed and strong optimization ability. In SSA, the solution of the optimization problem is obtained by simulating the sparrow foraging process, which includes discoverer sparrows, follower sparrows, and predators.

During each iteration, the location of the discoverer is updated as below:

(3) $$X_{i,j}^{t+1}=\{\begin{array}{cc} X_{i,j}^{t}*\exp\left(\frac{-i}{\alpha *iter_{\max}}\right) & if\;R_{2}<ST\\ X_{i,j}^{t}+Q*L & if\;R_{2}\geq ST \end{array}$$

where t indicates the number of iterations, $X_{i,j}$ represents the location of the i-th sparrow at the j-th dimension, and $iter_{\max}$ is a constant with the largest number of iterations. α is a random number, α∈(0,1]. $R_{2}$ and ST represent the alarm value and the safety threshold, respectively, $\left(R_{2}\in \left[0,1\right]\right)$, (ST∈[0.5,1.0]). Q is a random number which obeys normal distribution. L shows a matrix that each element inside is 1.

When $R_{2}<ST$, it means that there are no predators around the foraging environment, and the discoverer can perform a wide range of search operations. When $R_{2}\geq ST$, it indicates that some sparrows have found predators and sent an alarm to other sparrows in the population. At this time, all sparrows need to quickly fly to other safe places for foraging.

The location update description of the follower is as follows:

(4) $$X_{i,j}^{t+1}=\{\begin{array}{cc} Q*\exp\left(\frac{X_{worst}^{t}-X_{i,j}^{t}}{i^{2}}\right) & if\;i>n/2\\ X_{P}^{t+1}+\left|X_{i,j}^{t}-X_{P}^{t+1}\right|*A^{+}*L & otherwise \end{array}$$

where $X_{P}$ is the optimal position occupied by the discoverers. $X_{worst}$ denotes the current worst location. n is the population number of sparrows. A represents a matrix that each element inside is randomly assigned 1 or −1, and $A^{+}=A^{T}{\left(AA^{T}\right)}^{-1}$.

When the sparrow population is aware of danger, it will make anti-predation behavior; the mathematical expression is as follows:

(5) $$X_{i,j}^{t+1}=\{\begin{array}{cc} X_{best}^{t}+\beta *\left|X_{i,j}^{t}-X_{best}^{t}\right| & if\;f_{i}>f_{g}\\ X_{i,j}^{t}+K*\left(\frac{\left|X_{i,j}^{t}\;-\;X_{worst}^{t}\right|}{\left(f_{i}\;-\;f_{w}\right)\;+\;\varepsilon }\right) & if\;f_{i}=f_{g} \end{array}$$

where $X_{best}$ is the current best location. β is the random parameter which obeys the normal distribution. K is a random number, K∈(−1,1). $f_{i}$ is the fitness value of the present sparrow. $f_{g}$ is the current best fitness values, and $f_{w}$ is the current worst fitness values. The fitness value is calculated by root mean square error (RMSE) of predicted dam data and original dam data. ε is the smallest constant so as to avoid the denominator to be zero.

The sparrows update the location according to Equations (3)–(5) until the maximum number of iterations is reached, and the global optimal BiLSTM parameter is determined where the sparrow has the highest fitness value. Then, the SSA-BiLSTM prediction model of dam effect quantity can be constructed by using the optimized model parameters.

### 2.3. SSA-BiLSTM Fusion Prediction Model

**Figure 3:**
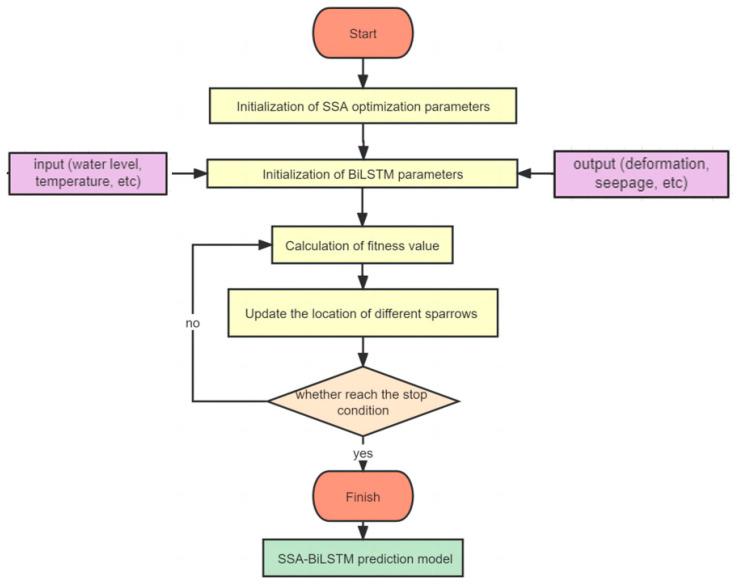
SSA-BiLSTM specific steps.

In BiLSTM neural network, four important model parameters (maximum number of iterations, initial learn rate, number of layers of hidden layers, number of hidden units) have a great impact on the accuracy of prediction results. Therefore, taking the four parameters in BiLSTM as the optimization variables of SSA. The specific steps of SSA-BiLSTM are as follows and shown in Figure 3.

(1) Initialization of SSA optimization parameters: SSA parameters includes sparrow population size, initial location, optimal location, and global optimal fitness. Rank the initial sparrow positions based on the fitness value. Select the first 20% as the discoverers and the rest as the followers.

(2) Optimization matrix: The search space matrix is formed based on the number of sparrows and optimization parameters (maximum number of iterations, initial learn rate, number of layers of hidden layers, and number of hidden units) and set the maximum number of iterations.

(3) Construction of prediction model: Taking the dam environmental quantity and effect quantity as the input and output set of BiLSTM, the prediction model of dam effect quantity based on BiLSTM is constructed.

(4) Parameter optimization: Update the location of the discoverer and follower according to Equation (3) to Equation (5), and recalculate the fitness values of different sparrows by substituting them into the BiLSTM model.

(5) Optimization finished: Determine whether the stop condition for the maximum number of iterations is satisfied. If yes, set the global optimal parameter group as the parameter of BiLSTM; If not, return to step (4).

## 3. Dam Safety Evaluation Model

Extreme loads such as earthquake and rainstorm have the characteristics of short duration and large variation. The dam mechanical parameters, such as dam deformation modulus and permeability coefficient, may change during extreme loads; these situations may cause the change of dam deformation and seepage behaviors under the same load. Therefore, in order to comprehensively evaluate the impact of extreme load on the long-term dam safety, it is necessary to establish the criterion of dam safety evaluation based on monitoring data.

The dam is subjected to the superposition of conventional load and extreme load during the dam whole life cycle. Conventional loads mainly include water pressure and temperature loads. There are different kinds of extreme loads, mainly earthquake and rainstorm. When the extreme load is over, the dam is subjected to conventional loads again. The dam monitoring data before and after the extreme load condition can effectively be used to compare and evaluate the impact of extreme load on dam safety by analyzing whether the change law of dam effect quantity has changed significantly.

The steps for obtaining evaluation information are as follows: (a) The monitoring data of the dam before extreme load conditions are input into the constructed SSA-BiLSTM model to establish the prediction model of dam effect quantity before extreme load conditions. (b) Based on the constructed prediction model, which accurately quantified the input-output relationship of the dam before the occurrence of extreme load, the environmental quantity after the end of extreme load is input into the constructed model to obtain the predicted value of dam effect quantity after the end of extreme load. (c) Calculate the error series between the measured value and the predicted value after the extreme load.

When the change law of dam effect quantity after the extreme load condition is similar to the duration before the extreme load condition, it indicates that the extreme load has no obvious impact on the dam safety. However, when the accuracy of the prediction model greatly decreases after the extreme load, it indicates that the safety status of the dam may change due to the influence of the extreme load. It needs to be further checked by on-site inspection whether the dam has cracks, seepage problems or other risk situation.

Except for the duration of extreme load, the dam is affected by conventional load before and after the occurrence of extreme load. According to the dam safety monitoring theory, under the influence of conventional loads, such as water pressure and temperature, the dam’s effect quantity y (deformation, seepage, etc.) can be divided into water pressure component, $y_{H}$; temperature component, $y_{T}$; and time-dependent component, $y_{\theta }$; according to the influencing factors; and the expression is as follows:

(6) $$y=y_{H}+y_{T}+y_{\theta }$$

The specific expressions of each component are shown in the literature [31,32]. According to Equation (6), the input and output variables of the SSA-BiLSTM model can be determined.

**Figure 4:**
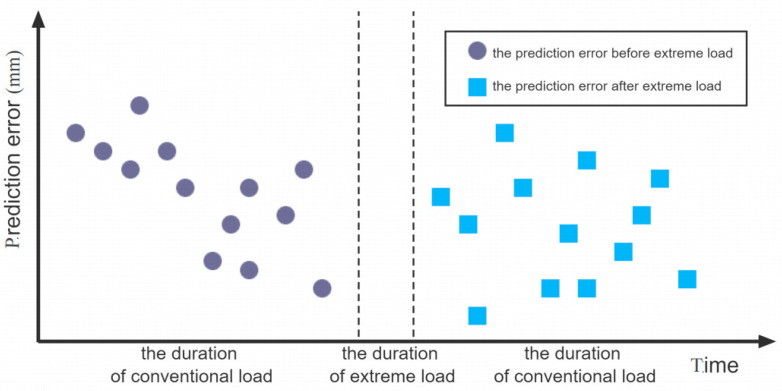
Error distribution of prediction model before and after extreme load.

Finally, the safety evaluation model based on the Mann–Whitney U-test method is proposed. U-test is a non-parametric data test method commonly used in statistics to evaluate whether two independent data samples come from the same population [33]. If the effect quantity, y, of the dam changes greatly after the end of the extreme load, the error of the predicted model before and after the extreme load will have differed significantly. the U-test method can effectively detect this situation. The test principle is as follows:

Taking the date of extreme load as the demarcation point, the errors of the prediction model are divided into two groups in Figure 4, U statistics are expressed as follows:

(7) $$U=\frac{\left|Y_{1}-Y_{2}\right|}{\sqrt{S_{1}^{2}/n_{1}+S_{2}^{2}/n_{2}}}$$

where $Y_{1}$ and $Y_{2}$ are the mean value of the error series of the SSA-BiLSTM model before and after the extreme load conditions; $n_{1}$ and $n_{2}$ are the number of the two samples; $S_{1}$ and $S_{2}$ are the standard deviation of the two samples.

Based on the predetermined degree of confidence α, the $U_{\alpha }$ can be checked out from the normal distribution table. The evaluation index of whether the dam safety behavior changes after extreme load are as follows:

(1) $U<U_{\alpha }$, The extreme load has little impact on the safety state of the dam, and the dam safety state is consistent with that before the occurrence of extreme load.
(2) $U\geq U_{\alpha }$, The extreme load has significant impact on the safety state of the dam, and the dam safety state is not consistent with the duration before the occurrence of extreme load, the dam may in the unsafe operation state.

According to the Guidelines for Dam Safety Evaluation (SL258-2000), issued by the Ministry of Water Resources of China, dam safety evaluation includes structural safety evaluation, seepage safety evaluation, etc. The evaluation methods mainly include on-site inspection, instrument testing, monitoring data analysis, and expert scoring. Among them, monitoring data analysis is the most objective and accurate evaluation method in dam safety evaluation, and its analysis results are used as the core basis for the evaluation results. Therefore, the dam safety evaluation method based on monitoring data proposed in this paper can provide scientific basis for the safety evaluation of dams subjected to extreme loads. It can derive the final evaluation result to integrate the results of on-site inspection and instrument testing to comprehensively evaluate the dam safety status.

## 4. Case Study

### 4.1. Project Overview

**Figure 5:**
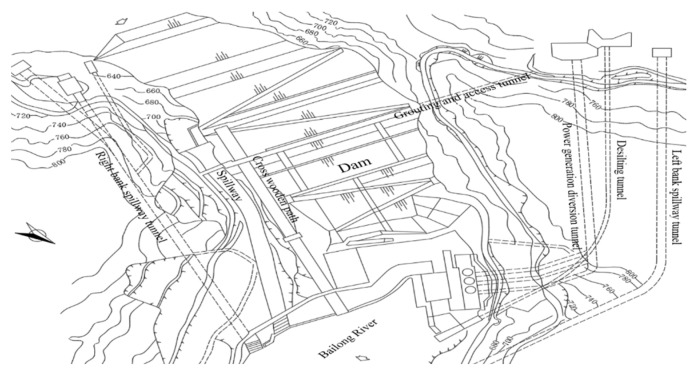
Vertical view of dam project.

**Figure 6:**
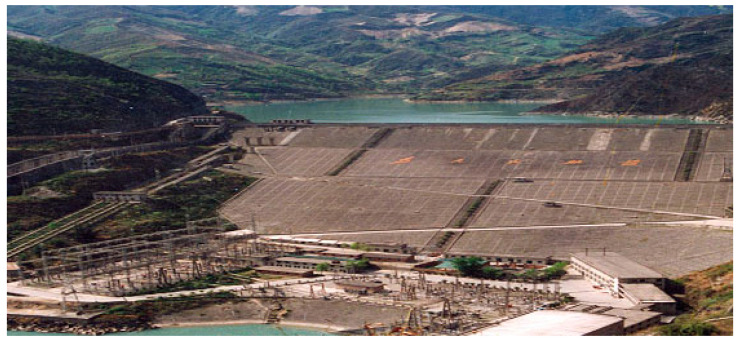
The general view of the dam.

An earth-rock dam is located in Bailong River, Gansu Province, China. The project performs an important role in the local power generation, flood control, and irrigation. The reservoir capacity and height of the project are 5.21 × 10^8^ m^3^ and 101.80 m, respectively. The layout of the dam is shown in Figure 5, and the general view of the dam is shown in Figure 6.

**Figure 7:**
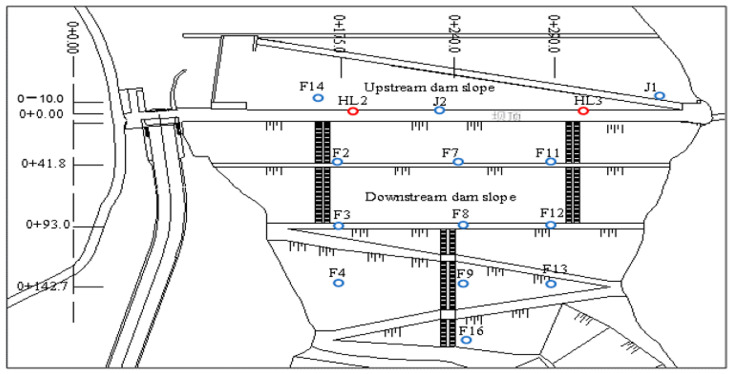
The layout of monitoring system.

In order to monitor the safety operation of the dam, a comprehensive monitoring system is arranged in the project. In addition to environmental quantity monitoring items, such as water level, rainfall, and temperature, effect quantity monitoring items are also included, which mainly include deformation and seepage. The layout of monitoring system is shown in Figure 7. The numbers in Figure 7 represent the position coordinates with the unit of meters.

### 4.2. Contents of Dam Safety Evaluation

Dam safety evaluation includes deformation safety evaluation, seepage safety evaluation, etc. In this study, the deformation safety evaluation is taken as an example, which is the most important effect quantity, and the seepage safety evaluation process is similar to the deformation evaluation.

This dam project was built in 1997 and has been in operation for 25 years. The dam has experienced not only conventional loads but also some extreme loads during operation. The typical extreme loads experienced by the project are shown in Table 1. In 2017, a sudden heavy rainfall in summer lasted for a week, which had a significant impact on the dam and its surroundings. In 2008 and 2010, the project encountered two strong earthquakes, of which the Wenchuan earthquake was widely concerned because of its large magnitude and strong destructive power.

As the Wenchuan earthquake caused serious risk situations for this dam project. This study takes the Wenchuan earthquake event as an example to analyze the changing trend of dam deformation safety state after the earthquake. The original data, such as dam environmental quantity data and deformation monitoring data, are all provided by the dam management department.

### 4.3. Process of Dam Safety Evaluation

(1) Construction of SSA-BiLSTM model before extreme load

*a. Data Selection*

The first step of safety evaluation is to construct a high-precision SSA-BiLSTM prediction model based on the long-term dam monitoring data before the earthquake. Three typical measured points, D10-2, D11-2, and D13-2, which are close to the dam crest, are selected for analysis. The long-term measured data before Wenchuan earthquake from 24 November 2006 to 8 May 2008 is selected for the modeling data.

Totally 85% of the modeling data are used as training data, and the remaining 15% are used as test data to test the prediction accuracy of the model. The selection of modeling data is shown in Table 2.

*b. Input-output selection*

**Figure 8:**
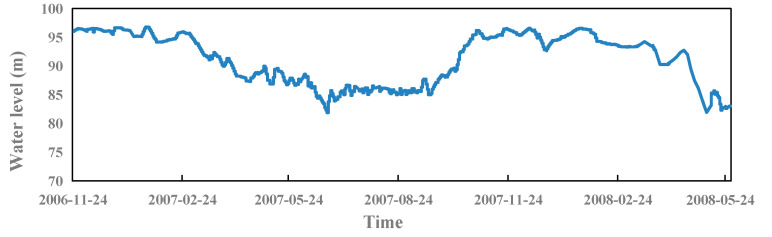
Process line of water level.

The second step is to determine the input and output relationship of the SSA-BiLSTM model. According to dam deformation monitoring theory, the model has 16 input factors, which divided into three parts: (1) water pressure component: *X*_1_ = (*H* − *H*_0_), *X*_2_ = (*H* − *H*_0_)^2^, *X*_3_ = (*H* − *H*_0_)^3^, and *X*_4_ = (*H* − *H*_0_)^4^. These factors are the direct influence of water pressure. The second input of water pressure component is the indirect hysteresis effect of water pressure, which includes four factors: *X*_5_ = (*H*_1–3_ − *H*_0_), *X*_6_ = (*H*_4–10_ − *H*_0_), *X*_7_ = (*H*_11–30_ − *H*_0_), and *X*_8_ = (*H*_31–60_ − *H*_0_). *H*_m-n_ is the average water level from the m-th day to the n-th day. *H*_0_ is the water level corresponding to the initial monitoring date. The water level process line is shown in Figure 8.

**Figure 9:**
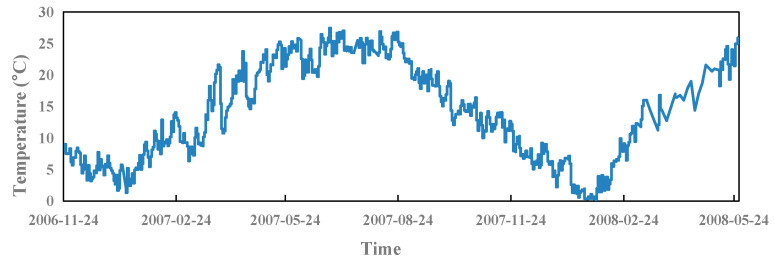
Process line of environmental temperature.

When there is no thermometer embedded in the dam, it generally selects different hysteresis factors of environmental temperature data as the input of temperature components. It generally includes six parts: *X*_9_ = *T*, *X*_10_ = *T*_1–3_, *X*_11_ = *T*_3–5_, *X*_12_ = *T*_5–20_, *X*_13_ = *T*_20–60_, and *X*_14_ = *T*_60–90_. *T*_m–n_ is the average environmental temperature from the m-th day to the n-th day. The environmental temperature process line is shown in Figure 9.

In addition, time-dependent component is the irreversible deformation related to the time. The input of time-dependent component is generally selected: *X*_15_ = θ − θ_0_, *X*_16_ = lnθ − lnθ_0_ (θ = t/100, θ_0_ = t_0_/100).

**Figure 10:**
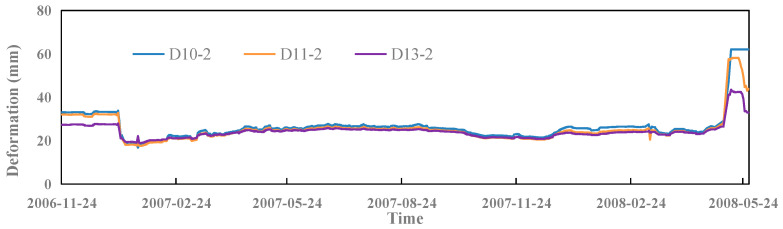
Process line of dam deformation of three typical measured points.

The output of the model is the deformation value of the dam measured point. The deformation process of the three typical measured points, D10-2, D11-2, and D13-2, is shown in Figure 10.

*c. Model parameter setting*

Before establishing the BiLSTM model, four parameters are needed: maximum number of iterations, initial learning rate, number of layers of hidden layers, and number of hidden units. In BiLSTM neural network, the selection of parameters has a great influence on model training and prediction accuracy. In order to obtain the optimal parameters of the BiLSTM model, it is necessary to use the SSA optimization algorithm to optimize the parameters. The initial SSA parameters are set as follows: The total number of sparrows in SSA is 30, with 20% of discoverers and 80% of followers. The hidden layer neuron number search range is [10, 500], the iteration number search range is [10, 200], and the learning rate search range is [0.001, 0.2].

By using the SSA optimization algorithm, the optimal model parameters of BiLSTM are: 120(maximum number of iterations), 0.05(initial learn rate), 1(number of layers of hidden layers), and 64(number of hidden units).

*d. Comparison model*

In order to verify the prediction accuracy of the proposed SSA-BiLSTM model, multiple comparison models need to be selected. There are two principles for selecting comparison models. One is to verify the prediction advantages of single BiLSTM model. Therefore, BP neural network, LSTM deep learning neural network are selected as the comparison models to analyze the prediction performance of different single prediction models. Another principle is to verify the improvement of the optimization algorithm on the prediction accuracy of BiLSTM; GA-BiLSTM is selected for comparison, and Genetic Algorithm (GA) is a commonly used optimization algorithm.

*e. Model prediction results*

**Figure 11:**
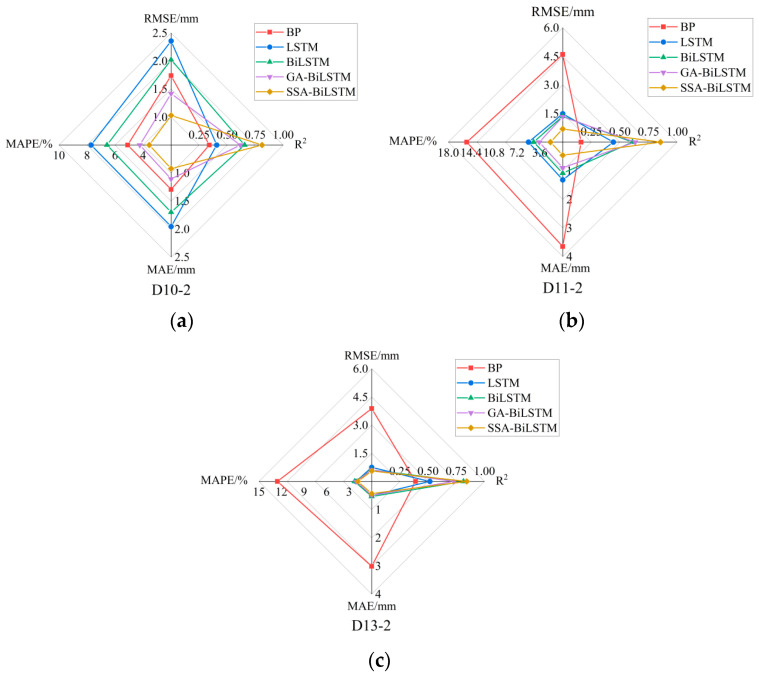
(**a**) The prediction index of different models of D10-2. (**b**) The prediction index of different models of D11-2. (**c**) The prediction index of different models of D13-2.

After obtaining the model parameter, it is necessary to validate the prediction effect of the optimized SSA-BiLSTM model through predicting the value of the test data. The prediction effects of the test data using different models are shown in Figure 11. Figure 11a–c show the prediction accuracy results of each model at three measurement points respectively. Radar chart is often used to evaluate the accuracy of prediction models. The commonly used accuracy indicators include mean absolute error (MAE), mean absolute percentage error (MAPE), root mean square error (RMSE), and R-Square (R^2^). Radar chart overcomes the problem that scatter chart can only show two indicators to represent the accuracy of the model and can comprehensively measure the accuracy of different prediction models.

It can be found from Figure 11 that even the five prediction models have excavated the law of data change, but BP model and LSTM model have relatively lower accuracy in the prediction of test data due to the simpler neural network structure than BiLSTM model, which is an improved LSTM model. The MAPE and MAE values of the BiLSTM prediction model at the three measuring points are 13.88% and 7.77%, respectively, lower than those of LSTM on average. GA-BiLSTM model integrates the advantages of BiLSTM and parameter optimization, which performs better in the prediction than BiLSTM. Through the comparison between GA-BiLSTM and SSA-BiLSTM, it can be found that the optimization effect of SSA is better than that of GA optimization algorithm, which shows that SSA can improve the global optimization ability of super parameters and improve the accuracy of BiLSTM prediction model.

(2) The prediction results after extreme load

**Figure 12:**
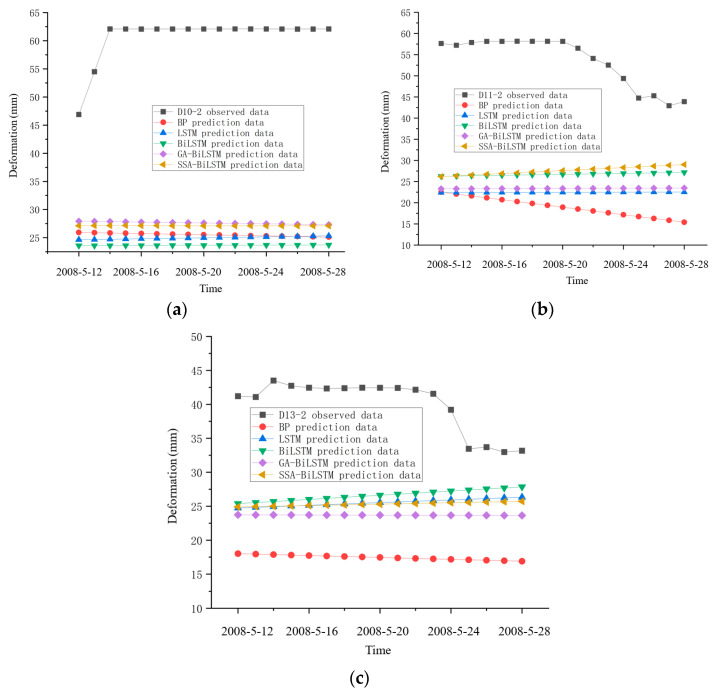
(**a**) The prediction result of prediction models after earthquake of D10-2. (**b**) The prediction result of prediction models after earthquake of D11-2. (**c**) The prediction result of prediction models after earthquake of D13-2.

The important content to evaluate the safety status of the dam after the earthquake is to study whether the deformation law of the dam changes. First, the constructed SSA-BiLSTM model and the four compared models are used to construct the prediction model before the earthquake. Then, input the environmental quantity data (water pressure, temperature, etc.) of the dam after the earthquake into the constructed prediction models. Finally, outputting the results of the observed and predicted values of dam deformation which shown in Figure 12. Figure 12a–c show the prediction results of each model at three measurement points respectively. Since the short-term dam safety after the earthquake is particularly important, the figure shows the output data from 12 May to 28 May.

Figure 12 shows that the prediction accuracy of all the prediction model has declined significantly after the earthquake, and the deviation between the predicted value and the observed value of the three-measurement point exceeds 15 mm. The predicted value and the observed value have deviated significantly, which indicates that the predicted change law is not consistent with that before the earthquake.

(3) The dam safety evaluation after the extreme load

**Figure 13:**
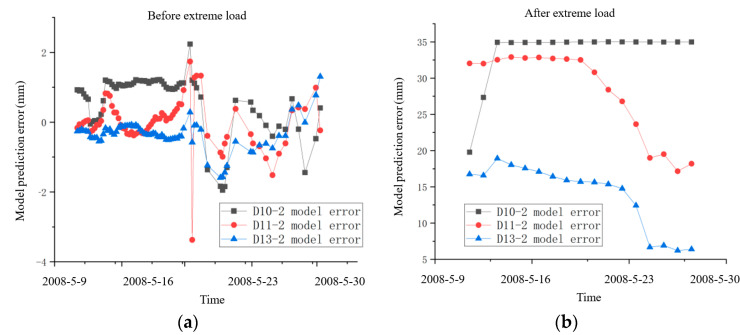
(**a**) SSA-BiLSTM prediction error before the earthquake. (**b**) SSA-BiLSTM prediction error after the earthquake.

According to the proposed dam safety evaluation model, the key to dam safety evaluation after extreme load is to analyze whether the changing law of dam effect quantity changes after the extreme load. It can be found in Figure 13a that before the occurrence of extreme load, the prediction accuracy of the SSA-BiLSTM model is high, and the maximum error of the model is within 3.5 mm. The prediction error of the model fluctuates steadily around the zero point, indicating that the prediction model can accurately simulate the deformation law of the dam before the occurrence of extreme load. Figure 13b shows that when the extreme load ends, the deviation between the observed value and the predicted value at each measuring point increases significantly, indicating that the constructed prediction model cannot accurately simulate the deformation law of the dam after the extreme load ends, and the deformation law of the dam has changed greatly. Therefore, the safety evaluation model needs to be used to quantitatively evaluate the safety status of the dam.

According to the safety evaluation criteria shown in Equation (7), it needs to first calculate the mean value and variance of the error series of SSA-BiLSTM prediction model before and after the occurrence of extreme load. Then, it needs to calculate the U value of the two-error series according to the U-test principle. The calculation results are shown in Table 3. When using U-test to determine whether two series obey the same distribution law, it is necessary to select the corresponding confidence level α, it generally selects α=0.05 [34]. According to the normal distribution table, the corresponding critical $U_{\alpha }$ value is 1.96. The U value of the three measuring points is significantly greater than the $U_{\alpha }$, indicating that the deformation law of the three measuring points has changed significantly after the occurrence of extreme load. Therefore, Figure 13 and Table 3 all show that the dam is in unsafe state after the end of extreme load. The dam shall be reinforced in time to prevent risk during the operation period.

(4) The validation of safety evaluation result

**Figure 14:**
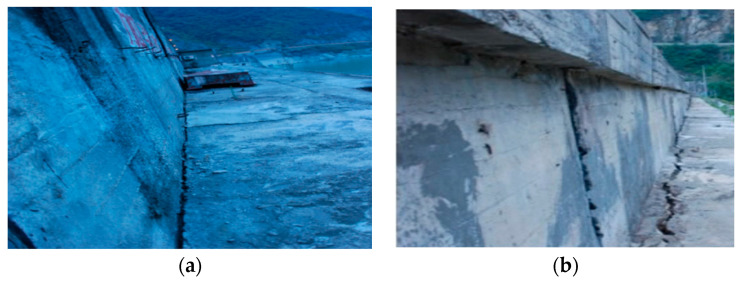
(**a**) Image of dam earthquake damage of upstream wave wall. (**b**) Image of dam earthquake damage of downstream wave wall.

According to the Guidelines for Dam Safety Evaluation (SL258-2000), issued by the Ministry of Water Resources of China, the dam management department evaluated the safety status of the dam after the earthquake through on-site inspection method. According to the on-site inspection result, obvious changes have taken place in some parts of the dam which shows in Figure 14 [35], it indicates that continuous cracks are generated on the upstream and downstream wave walls of the dam which shows in Figure 14a,b respectively. The dam crest has obviously deformation towards the upstream direction, and the maximum displacement occurs on the left of the dam crest is 157.5 mm. The downstream slope of the dam has deformation towards the downstream direction with the maximum deformation of 121.2 mm. The D10-2, D11-2 and D13-2 measuring points are all located at the downstream part of the dam crest. The initial deviation of the observed values and predicted values of the three measuring points after the earthquake exceed 15 mm, indicating that the dam deformation caused by the earthquake is the main reason for the large deviation of the observed values and predicted values, and the dam has suffered obvious earthquake damage.

According to the management report of the dam engineering department after the earthquake, the engineering department has carried out grouting reinforcement treatment on the joints between the wave wall and the core wall on the dam crest, which also verified the accuracy of the proposed safety assessment results. Therefore, the proposed method can provide technical support for the reinforcement work after the project encounters extreme load. Through the safety evaluation analysis of each part in the dam based on the monitoring data, the damage degree of different parts can be quantitatively evaluated, so as to carry out differential reinforcement work to save project investment.

## 5. Conclusions

The dam safety evaluation after extreme load is of great significance to dam safety management. This article combines artificial intelligence algorithms, optimization algorithms, and data validation theory to propose a dam safety evaluation method based on SSA-BiLSTM and U-test. First, the theory of SSA-BiLSTM prediction model is introduced in detail. Second, the application steps of the SSA-BiLSTM fusion model in data prediction of dam monitoring data is displayed. Then, the U-test method is introduced to construct the evaluation index to evaluate whether the change law of dam effect quantity has changed significantly after the occurrence of extreme load. Finally, it proposes an evaluation method for the dam safety state. The following results are obtained through case analysis:

(1) In view of the problem that there are many kinds of extreme loads, and the process simulation methods are different and complex, the dam safety evaluation under extreme load is converted into the evaluation before and after the occurrence of extreme load. Since the operation conditions before and after the occurrence of extreme load are conventional, the influencing factors and mechanism of dam effect have been fully studied. A unified safety evaluation model can be established without considering the difference of extreme loads.
(2) A dam safety evaluation fusion model based on SSA-BiLSTM, and U-test is proposed. The problem of manual parameter adjustment is effectively solved by optimizing the parameters of BiLSTM through SSA algorithm. Whether the prediction accuracy of the prediction model changes before and after the occurrence of extreme load is evaluated through U-test quantitative evaluation, then the safety state of the dam is evaluated.
(3) Taking an earth-rock dam in China as an example, the earth-rock dam experienced the Wenchuan 8.0 earthquake in 2008. The prediction model results show that SSA-BiLSTM can accurately model the deformation law of the dam before the earthquake. Through the U-test method, it is found that the accuracy of the prediction model has changed significantly after the earthquake, and the dam is in a dangerous operation state. The effectiveness and accuracy of the analysis results are verified by comparing with the on-site detection results of the management department. Therefore, this method can be used as a new way for dam safety evaluation, not only for extreme load problems, but also for dam safety evaluation when the dam is in normal operation.
(4) This article adopts a combination of SSA optimization algorithm and BiLSTM artificial intelligence algorithm. Due to the rapid development of optimization algorithms and artificial intelligence algorithms, it can study different combinations of optimization algorithms and artificial intelligence algorithms in the future research. Moreover, this article analyzes the safety evaluation after extreme loads, the application of the method can be expanded to dam safety evaluation under normal operating conditions in the future research.

## Figures and Tables

**Table 1:** Typical extreme loads condition of the dam.

Extreme Load Condition	Time	Grade
Earthquake	2008.5.12	Magnitude scale (8.0)
Earthquake	2010.4.14	Magnitude scale (7.1)
Rainstorm	2017.8.20	Daily rainfall of 127 mm

**Table 2:** Selection of modeling data.

Measured Point	Training Data	Number	Test Data	Number
D10-2	2006.11.24~2008.1.16	412	2008.1.17~2008.5.8	73
D11-2	2006.11.24~2008.1.16	412	2008.1.17~2008.5.8	73
D13-2	2006.11.24~2008.1.16	412	2008.1.17~2008.5.8	73

**Table 3:** The U-test calculation results of the prediction model error series.

Monitoring Point	Mean Value of the Error Series before Extreme Load (mm)	Variance of the Error Series before Extreme Load (mm)	Mean Value of the Error Series after Extreme Load (mm)	Variance of the Error Series after Extreme Load (mm)	U Value
D10-2	0.61	0.83	33.62	3.90	34.74
D11-2	−0.02	0.69	28.03	5.85	19.72
D13-2	−0.35	0.43	13.97	4.33	13.60

## Data Availability

Data are available upon reasonable request.
